# Response of leaf stoichiometry of *Potentilla anserina* to elevation in China's Qilian Mountains

**DOI:** 10.3389/fpls.2022.941357

**Published:** 2022-09-26

**Authors:** Xiaofang Zhang, Qi Feng, Jianjun Cao, Asim Biswas, Haohai Su, Wei Liu, Yanyan Qin, Meng Zhu

**Affiliations:** ^1^Key Laboratory of Ecohydrology of Inland River Basin, Northwest Institute of Eco-Environment and Resources, Chinese Academy of Sciences, Lanzhou, China; ^2^University of Chinese Academy of Sciences, Beijing, China; ^3^College of Geography and Environmental Science, Northwest Normal University, Lanzhou, China; ^4^School of Environmental Sciences, University of Guelph, Guelph, ON, Canada; ^5^Qilian Mountains Eco-Environment Research Center in Gansu Province, Lanzhou, China; ^6^Key Laboratory of Land Surface Process and Climate Change in Cold and Arid Regions, Northwest Institute of Eco-Environment and Resources, Chinese Academy of Sciences, Lanzhou, China

**Keywords:** plant growth strategies, plant environmental adaptability, leaf traits, mountainous regions, nutrient limitation

## Abstract

Plants adapt to changes in elevation by regulating their leaf ecological stoichiometry. *Potentilla anserina* L. that grows rapidly under poor or even bare soil conditions has become an important ground cover plant for ecological restoration. However, its leaf ecological stoichiometry has been given little attention, resulting in an insufficient understanding of its environmental adaptability and growth strategies. The objective of this study was to compare the leaf stoichiometry of *P. anserina* at different elevations (2,400, 2,600, 2,800, 3,000, 3,200, 3,500, and 3,800 m) in the middle eastern part of Qilian Mountains. With an increase in elevation, leaf carbon concentration [(C)_leaf_] significantly decreased, with the maximum value of 446.04 g·kg^−1^ (2,400 m) and the minimum value of 396.78 g·kg^−1^ (3,500 m). Leaf nitrogen concentration [(N)_leaf_] also increased with an increase in elevation, and its maximum and minimum values were 37.57 g·kg^−1^ (3,500 m) and 23.71 g·kg^−1^ (2,800 m), respectively. Leaf phosphorus concentration [(P)_leaf_] was the highest (2.79 g·kg^−1^) at 2,400 m and the lowest (0.91 g·kg^−1^) at 2,800 m. The [C]_leaf_/[N]_leaf_ decreased with an increase in elevation, while [N]_leaf_/[P]_leaf_ showed an opposite trend. The mean annual temperature, mean annual precipitation, soil pH, organic carbon, nitrogen, and phosphorus at different elevations mainly affected [C]_leaf_, [N]_leaf_, and [P]_leaf_. The growth of *P. anserina* in the study area was mainly limited by P, and this limitation was stronger with increased elevation. Progressively reducing P loss at high elevation is of great significance to the survival of *P. anserina* in this specific region.

## Introduction

Ecological stoichiometry focuses on the balance of multiple elements in ecological interactions and processes, such as energy flow and nutrient cycling, and has become an important topic of research in recent years in ecology and biology (Moe et al., 2005; Sistla and Schimel, 2012; Yang et al., 2019; Zhu et al., 2020). It is an important tool to reveal organisms' responses to external disturbances and nutrient supply balance mechanisms in ecosystems (Zeng et al., 2016), mainly by analyzing changes in carbon (C), nitrogen (N), and phosphorus (P) (Cao et al., 2020), which are well-known as basic elements constituting plants and are closely linked to plant photosynthesis, respiration, and various ecosystem functions (Elser et al., 2010; Marschner and Marschner, 2012; Wang et al., 2015; Yan et al., 2016; Croft et al., 2017).

Leaves are the most active and primary photosynthetic plant organ (Elser et al., 2010; Yang et al., 2019), their size and structure exhibit a tradeoff between the support cost and photosynthetic returns during plant adaptation to environmental changes (Shi et al., 2020, 2022; Guo et al., 2021; Li et al., 2022a,b), and leaf stoichiometry can reflect the tradeoff formed in this evolution from the angle of leaf chemical elements and its spatio-temporal variations (Baxter and Dilkes, 2012; Cao et al., 2020; Zhu et al., 2020). Leaf C/N and C/P were widely accepted as effective indicators of plants' N and P use efficiency and growth rate, and their lower values indicated lower nutrient utilization efficiency and higher plant growth (Weidner et al., 2015; Sun et al., 2017; Cao et al., 2020; Zhang et al., 2020). Leaf N/P can reflect plant nutrient states and limitations (Tao et al., 2016). However, there remain questions about the N/P thresholds assessing plants' nutrient limitation during growth due to multiple factors (Crowley et al., 2012). For example, according to the study of Koerselman and Meuleman (1996) on wetland plant communities, when leaf N/P < 14, plant growth was limited by N; 14 ≤ leaf N/P ≤ 16, plant growth has the common limit of N and P; and leaf N/P > 16, plant growth was limited by P, while the study of Güsewell (2004) on terrestrial plant communities found that when leaf N/P < 10, plant growth was limited by N; 10 ≤ leaf N/P ≤ 20, plant growth has the common limit of N and P; and leaf N/P > 20, plant growth was limited by P. Nevertheless, leaf N/P is still considered to be a valuable tool for assessing potential patterns in nutrient limitation across broad landscapes (Crowley et al., 2012).

Relationships between leaf stoichiometry and environmental factors, including soil nutrients and geographical and climatic factors, were widely explored at various scales including regional and global scales (e.g., McGroddy et al., 2004; Reich and Oleksyn, 2004; Sardans et al., 2011; Du et al., 2017; Tian et al., 2018; Qin et al., 2021). Elevation is a crucial factor and can affect plant leaf stoichiometry by altering the combination of heat and water, soil properties, and vegetation community composition (Bo et al., 2020; Liu et al., 2021). However, there is little consensus on leaf stoichiometric characteristics as they change along an elevation gradient. Some studies observed that leaf C concentration [(C)_leaf_] increased but leaf N [(N)_leaf_] and leaf P concentration [(P)_leaf_] decreased with an increase in elevation (e.g., Zhao et al., 2014, 2018; Bo et al., 2020), while other studies reported an opposite trend (e.g., Macek et al., 2009; Li et al., 2018). Therefore, the response of plant leaf ecological stoichiometry to elevation should be investigated at regional and species-specific levels. Situated in the transition zone between the Qinghai–Tibetan Plateau and the arid region of northwestern China, the Qilian Mountains preserve a wide variety of plant species (Gui et al., 2020; Wang et al., 2022) and serve as an ideal region to study the impact of elevation on leaf stoichiometry of different plant species (Cao et al., 2020; Qin et al., 2021). *Potentilla anserina* L., a typical forb, is widely distributed in the Qilian Mountains. Due to its wide ecological amplitude and vegetative reproduction ability, *P. anserina* is an important species of degraded grasslands or secondary succession lands and is considered a prime species for ecological restoration in alpine regions (Sheng et al., 2004). Previous studies mainly focused on *P. anserina*'s chemical components (Zhao et al., 2020), pharmacological action (He et al., 2021), nutrient reabsorption (Li et al., 2020), and medicinal value (Cheng J. et al., 2021); information on its leaf stoichiometry remains unavailable. Exploring the leaf stoichiometry and growth nutrient limitation of *P. anserina* is of great significance for the ecological restoration and construction in the Qilian Mountains. It was found that the leaf stoichiometry of many dominant or common species in the Qilian Mountains was affected by elevation. For example, with increasing elevation, [C]_leaf_ of *Oxytropis ochrocephala* first increased and then decreased, while [N]_leaf_ and [P]_leaf_ of the same species showed a trend of general increase in the identified soil organic carbon (SOC), the ratio of SOC to soil total phosphorus (STP), and the mean annual temperature (MAT) as dominant factors (Cao et al., 2020). [C]_leaf_ and [P]_leaf_ of *Potentilla fruticosa* first decreased and then increased with an increase in elevation, and SOC, STP, and soil pH were the main factors influencing the leaf stoichiometry (Qin et al., 2022). We hypothesized that elevation may also significantly influence leaf stoichiometry of *P. anserina* and leaf stoichiometry of *P. anserina* could be influenced by soil nutrients, MAT, and MAP. Previous studies have proposed that the soil P content of the Qilian Mountains was relatively low (Xu et al., 2018, 2019). Therefore, we hypothesized that P could be a limiting nutrient for *P. anserina* growth in this region.

This study attempted to: (i) explore how *P. anserina* changed leaf C/N/P stoichiometry to adapt to environmental changes along elevation (2,400–3,800 m) in the Qilian Mountains, (ii) identify the underlying mechanism of the effect of elevation on leaf C/N/P stoichiometry by building structural equation models, and (iii) determine the key nutrients limiting the growth of *P. anserina* in the Qilian Mountains.

## Materials and methods

### Study area

A field study was conducted at the Dayekou River Basin and the Haibei Alpine Meadow Ecosystem Research Station in the Middle East of the Qilian Mountains (93°30′-103°30′ E, 36°30′-39°30′ N). The Qilian Mountains ranging from 2,000 to 5,500 m elevation belong to a typical plateau continental climate, with MAT and MAP of −0.4°C and 405 mm, respectively, from 2000 to 2015. Grasslands with *Carex tristachya, Stipa przewalskii, Leymus secalinus*, and *Polygonum viviparum* dominate the southern slopes, while Qinghai spruce (*Picea crassifolia* Kom.) forests dominate the northern slopes. Soils were classified as Haplic Kastanozems and Haplic Phaeozems on the southern and northern slopes, respectively (IUSS Working Group World Reference Base for Soil Resources, 2014).

### Field sampling

**Figure 1:**
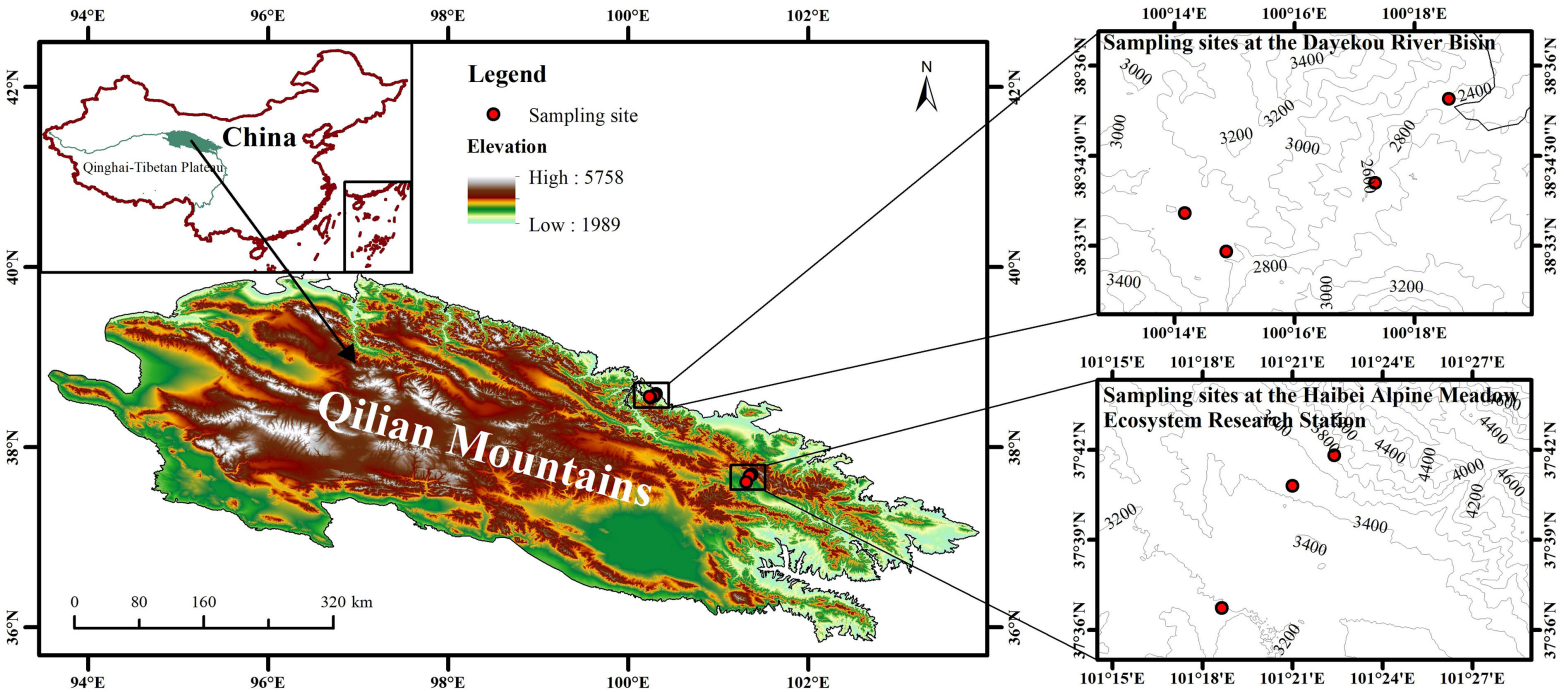
Sampling sites across elevations (2,400, 2,600, 2,800, 3,000, 3,200, 3,500, and 3,800 m) in the study area.

In August and September 2018, samples of healthy fresh leaves of *P. anserina* were collected from the following seven elevations: 2,400, 2,600, 2,800, 3,000, 3,200, 3,500, and 3,800 m (Figure 1). Sampling at 2,400, 2,600, 2,800, and 3,000 m was conducted at the Dayekou River Basin, while sampling at 3,200, 3,500, and 3,800 m was conducted at the Haibei Alpine Meadow Ecosystem Research Station due to their distribution as well as accessibility of the sites. At each elevation, leaves were collected from three random plots (10 × 10 m) and then packed in paper bags. All the sampling plots were on the southern slopes with slope gradients between 27° and 33°. In each plot, three quadrats (1 × 1 m) were positioned evenly along a diagonal line. Soil samples from 0 to 0.10, 0.10 to 0.20, and 0.20 to 0.40 m were collected by using a 100-mm-diameter soil auger (Cao et al., 2020). A total of 21 sampling plots (seven elevations × three plots) and 63 sample quadrats (21 plots × three quadrats in each plot) were investigated.

### Leaf and soil analyses

The leaves were firstly oven-dried at 80°C for 24 h and then ground to determine [C]_leaf_, [N]_leaf_, and [P]_leaf_. Soil samples were air-dried and divided into two parts: One part was ground and sieved through a 100-mesh sieve for SOC, soil total nitrogen (STN), and STP determination, and the other part was ground and sieved through an 8-mesh sieve for soil pH determination. [C]_leaf_ (g kg^−1^) and SOC (g kg^−1^), [N]_leaf_ (g kg^−1^) and STN (g kg^−1^), and [P]_leaf_ (g kg^−1^) and STP (g kg^−1^) were measured using the potassium bichromate titrimetric method (Nelson and Sommers, 1982), the Kjeldahl method (Bremmer and Mulvaney, 1982), and the molybdate blue method (Olsen and Sommers, 1982), respectively. Soil pH was measured by the potentiometric method with a soil–water ratio of 2:5.

### Data analysis

MAT and MAP (Table 1) were calculated according to Zhao et al. (2005, 2006) as follows:


(1) $$\begin{array}{ll} MAT=20.96-5.49\times 10^{-3}ELEV-0.17LAT+8.9\\ \times & 10^{-3}{LONG,\;R}^{2}=0.98 \end{array}$$

(2) $$\begin{array}{l} MAP=1.68\times 10^{3}+0.12ELEV+12.41LAT\\ -75.26{LONG,\;R}^{2}=0.92 \end{array}$$

where *ELEV* is the elevation, *LAT* is the latitude, *LONG* is the longitude, and *R*^2^ is the regression coefficient.

**Table 1:** Sampling site coordinates, mean annual temperature (MAT), and precipitation (MAP) in the study area.

**Parameter**	**Elevation**						
	**2,400 m**	**2,600 m**	**2,800 m**	**3,000 m**	**3,200 m**	**3,500 m**	**3,800 m**
LONG	100°19′12″ E	100°17′18.5″ E	100°14′28″ E	100°14′26″ E	101°18′36″ E	101°21′00″ E	101°22′12″ E
LAT	38°35′24″ N	38°34′2.8″ N	38°33′9″ N	38°33′22″ N	37°36′36″ N	37°40′48″ N	37°41′24″ N
MAT (°C)	2.27	1.17	0.08	−1.02	−1.95	−3.61	−5.26
MAP (mm)	306.26	331.36	355.70	379.22	487.50	518.42	553.62

LAT, latitude; LONG, longitude; MAT, mean annual temperature; MAP, mean annual precipitation.

All data were described by their average value and standard error (SE). Soil properties in the 0–0.40 m soil layer were expressed as an average of values in the 0–0.10, 0.10–0.20, and 0.20–0.40 m soil layers. The fixed effect (elevation) and random effect (experimental plots) on leaf stoichiometry of *P. anserina* and soil properties were tested by fitting linear mixed models (LMMs) in R3.3.1 (*nlme* vegan). The differences in soil properties among elevations were detected using the one-way ANOVA, followed by the least significant difference test to perform the significance analysis at *P* < 0.05 in SPSS 22.0 (SPSS Inc., Chicago, IL, USA). Pearson's correlation analysis was conducted to explore the correlations between the leaf stoichiometry of *P. anserina* and the topography-induced climatic factors and soil properties in SPSS 22.0. Path analysis was used in the structural equation modeling (SEM) to evaluate the direct and indirect effects of soil properties on leaf stoichiometry of *P. anserina* in Amos 22.0 (IBM SPSS Inc., Chicago, IL, USA).

## Results

### Variations in leaf stoichiometry of *Potentilla anserina* and soil properties with elevation

**Figure 2:**
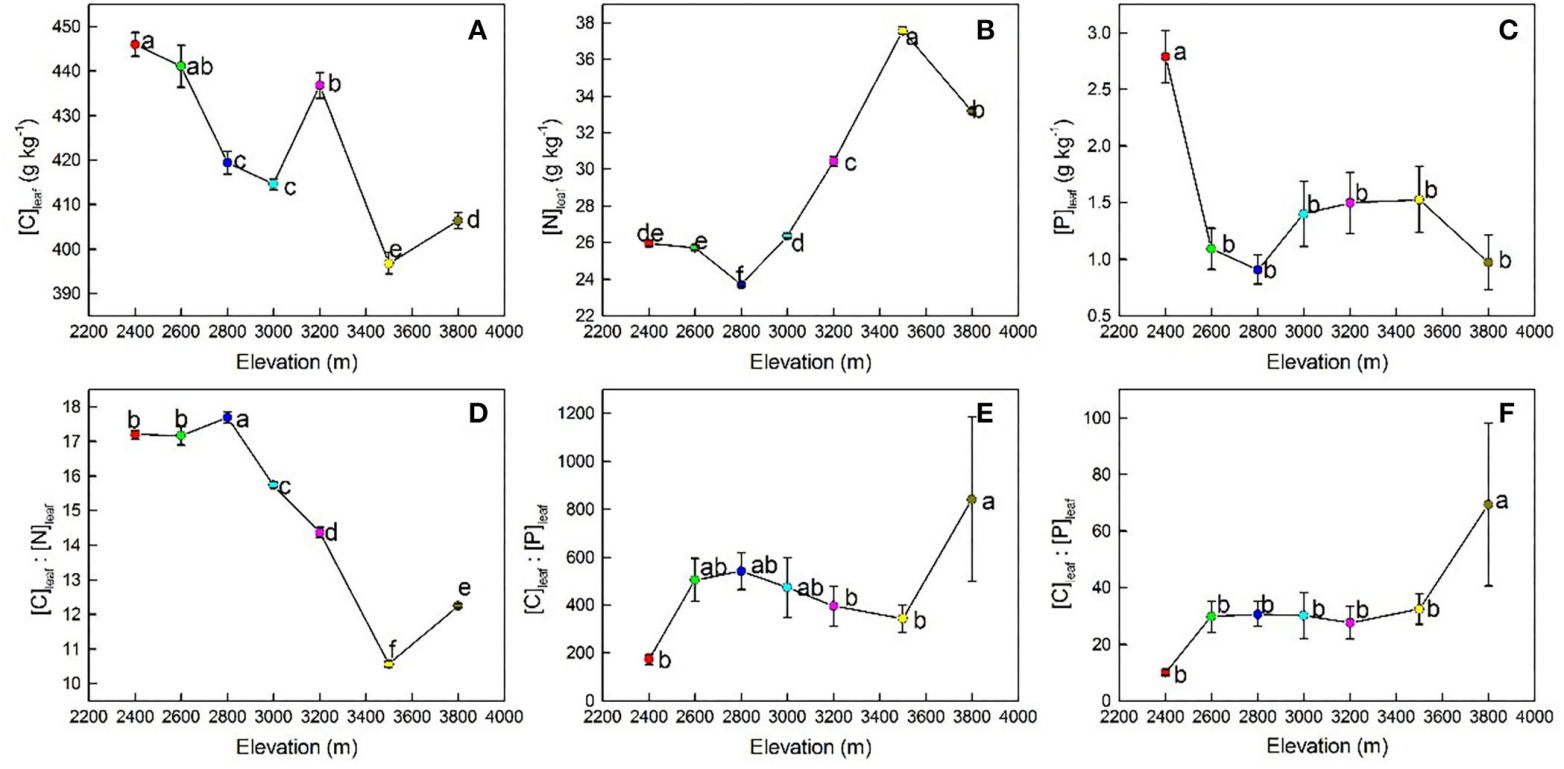
Changes in *Potentilla anserina*'s [C]_leaf_ **(A)**, [N]_leaf_ **(B)**, [P]_leaf_ **(C)**, and their ratios **(D–F)** with an increase in elevation. [C]_leaf_, leaf carbon concentration; [N]_leaf_, leaf nitrogen concentration; [P]_leaf_, leaf phosphorus concentration. Different lowercase letters indicate a significant difference in leaf stoichiometry and soil properties among elevations (*P* < 0.05).

The results of LMMs showed that leaf stoichiometries of *P. anserina* and soil properties were significantly affected by elevation (Table 2). With increasing elevation, [C]_leaf_ showed a generally decreasing trend (Figure 2A) and reached its maximum and minimum values at 2,400 m (446.04 ± 8.17 g kg^−1^) and 3,500 m (396.78 ± 7.38 g kg^−1^), respectively (Table 3). The [N]_leaf_ showed a generally increasing trend (Figure 2B), with its maximum and minimum values at 3,500 m (37.57 ± 0.64 g kg^−1^) and 2,800 m (23.71 ± 0.40 g kg^−1^), respectively (Table 3). The [P]_leaf_ decreased from 2,400 to 2,600 m elevation and reached the maximum and minimum values at 2,400 (2.79 ± 0.69 g kg^−1^) and 3,800 m (0.97 ± 0.72 g kg^−1^), respectively (Figure 2C, Table 3). However, there was no difference in [P]_leaf_ at different elevations above 2,600 m (Table 3). In contrast to [N]_leaf_, the [C]_leaf_/[N]_leaf_ showed a generally decreasing trend with an increase in elevation (Figure 2D), with a peak at 2,800 m (17.69 ± 0.46), and then reduced at 3,500 m (10.57 ± 0.27) (Table 3). The maximum and minimum values of [C]_leaf_/[P]_leaf_ were 842.36 ± 1,031.11 at 3,800 m and 173.03 ± 62.38 at 2,400 m, respectively. The [N]_leaf_/[P]_leaf_ showed a generally increasing trend with an increase in elevation (Figure 2F) and reached its maximum and minimum values at 3,800 m (69.40 ± 86.26) and at 2,400 m (10.05 ± 3.53), respectively (Table 3).

**Table 2:** Summary of the generalized linear mixed models for leaf stoichiometry and soil properties (0–0.40 m) (*n* = 9).

**Parameters**	**Fixed effect**			**Random effect**	**Fixed and**	***df***
	**(elevation)**			**(plot)**	**random effects**	
	***F***	***P***	***R^2^***	***P***	***R^2^***	
[C]_leaf_	44.27	**< 0.0001**	0.8107	0.9998	0.8107	6
[N]_leaf_	774.79	**< 0.0001**	0.9864	0.676	0.9868	6
[P]_leaf_	7.5862	**< 0.0001**	0.4053	0.4009	0.4479	6
[C]_leaf_/[N]_leaf_	341.89	**< 0.0001**	0.9703	0.8819	0.9707	6
[C]_leaf_/[P]_leaf_	2.4541	**0.0377**	0.1899	0.8675	0.2005	6
[N]_leaf_/[P]_leaf_	2.6180	**0.0282**	0.2021	0.9998	0.2021	6
pH	674.41	**< 0.0001**	0.9847	0.8551	0.9849	6
SOC	89.8473	**< 0.0001**	0.8870	0.2683	0.8980	6
STN	158.795	**< 0.0001**	0.9368	0.6607	0.9390	6
STP	10.3974	**< 0.0001**	0.5015	0.9999	0.5015	6
SOC/STN	70.067	**< 0.0001**	0.8715	0.9999	0.8715	6
SOC/STP	6.9747	**< 0.0001**	0.4030	0.9998	0.4030	6
STN/STP	7.6212	**< 0.0001**	0.4231	0.9413	0.4264	6

[C]_leaf_, leaf carbon concentration; [N]_leaf_, leaf nitrogen concentration; [P]_leaf_, leaf phosphorus concentration; SOC, soil organic carbon; STN, soil total nitrogen; STP, soil total phosphorus. *F*, Fisher's test; *P*, probability value; *df* , degree of freedom. Significant values are in bold.

**Table 3:** Variation of leaf stoichiometry of *Potentilla anserina* and soil properties (0–0.40 m) across elevations (mean ± standard error, *n* = 9).

**Parameter**	**Elevation**						
	**2,400 m**	**2,600 m**	**2,800 m**	**3,000 m**	**3,200 m**	**3,500 m**	**3,800 m**
[C]_leaf_ (g kg^−1^)	446.04 ± 8.17a	441.17 ± 14.22ab	419.37 ± 7.66c	414.62 ± 3.66c	436.79 ± 8.58b	396.78 ± 7.38e	406.34 ± 5.37d
[N]_leaf_ (g kg^−1^)	25.94 ± 0.55de	25.72 ± 0.46e	23.71 ± 0.40f	26.35 ± 0.38d	30.44 ± 0.82c	37.57 ± 0.64a	33.18 ± 0.41b
[P]_leaf_ (g kg^−1^)	2.79 ± 0.69a	1.09 ± 0.54b	0.91 ± 0.38b	1.40 ± 0.87b	1.50 ± 0.82b	1.53 ± 0.87b	0.97 ± 0.72b
[C]_leaf_/[N]_leaf_	17.20 ± 0.41b	17.17 ± 0.79b	17.69 ± 0.46a	15.74 ± 0.29c	14.36 ± 0.46d	10.57 ± 0.27f	12.25 ± 0.19e
[C]_leaf_/[P]_leaf_	173.03 ± 62.38b	505.25 ± 266.91ab	542.76 ± 230.80ab	472.84 ± 374.58ab	395.71 ± 249.70b	343.74 ± 174.94b	842.36 ± 1031.11a
[N]_leaf_/[P]_leaf_	10.05 ± 3.53b	29.75 ± 16.70b	30.66 ± 12.98b	30.13 ± 24.11b	27.62 ± 17.23b	32.42 ± 16.26b	69.40 ± 86.26a
pH	8.25 ± 0.02b	8.03 ± 0.07c	8.10 ± 0.09c	8.51 ± 0.09a	7.75 ± 0.16d	6.44 ± 0.16e	6.12 ± 0.10f
SOC (g kg^−1^)	25.52 ± 0.15d	48.99 ±2.52b	36.42 ± 2.57c	12.28 ± 0.61e	61.57 ± 3.15a	58.64 ± 1.65a	50.14 ± 1.65b
STN (g kg^−1^)	2.67 ± 0.03d	4.48 ± 0.17c	1.82 ± 0.13e	1.35 ± 0.10f	6.09 ± 0.25a	5.24 ± 0.13b	4.72 ± 0.13c
STP (g kg^−1^)	0.64 ± 0.01b	0.82 ± 0.05a	0.46 ± 0.01ce	0.32 ± 0.03de	0.48 ± 0.07ace	0.27 ± 0.05d	0.53 ± 0.12ace
SOC/STN	9.56 ± 0.05cd	10.94 ± 0.44bc	20.45 ± 1.18a	9.22 ± 0.35d	10.09 ± 0.21bcd	11.19 ± 0.08b	10.62 ± 0.24bcd
SOC/STP	39.9 2± 0.32d	60.48 ± 3.05cd	80.05 ± 6.90bcd	40.81 ± 3.96d	174.70 ± 45.29b	307.11 ± 79.17a	147.41 ± 33.46bc
STN/STP	4.18 ± 0.06d	5.60 ± 0.36cd	3.95 ± 0.30d	4.38 ± 0.32d	16.75 ± 3.91b	27.24 ± 6.90a	13.99 ± 3.21bc

[C]_leaf_, leaf carbon concentration; [N]_leaf_, leaf nitrogen concentration; [P]_leaf_, leaf phosphorus concentration; SOC, soil organic carbon; STN, soil total nitrogen; STP, soil total phosphorus. Different lowercase letters indicate a significant difference in leaf stoichiometry and soil properties among elevations (*P* < 0.05).

For soil properties, SOC and STN showed similar trends with their maximum values at 3,200 m (61.57 ± 3.15 g kg^−1^ and 6.09 ± 0.25 g kg^−1^, respectively), and minimum values at 3,000 m (12.28 ± 0.61 g kg^−1^ and 1.35 ± 0.10 g kg^−1^, respectively). The maximum and minimum values of STP were observed at 2,600 m (0.82 ± 0.05 g kg^−1^) and 3,500 m (0.27 ± 0.05 g kg^−1^), respectively. The maximum value of SOC/STN was observed at 2,800 m (20.45 ± 1.18), which was about two times that at other elevations. The values of SOC/STP and STN/STP at elevations ≥ 3,200 m were about two to seven times greater than those at elevations < 3,200 m. Soil pH at 3,000 m (8.51 ± 0.09) was the highest, while at 3,800 m (6.12 ± 0.10) it was the lowest.

### Environmental factors influencing leaf ecological stoichiometry of *Potentilla anserina*

**Figure 3:**
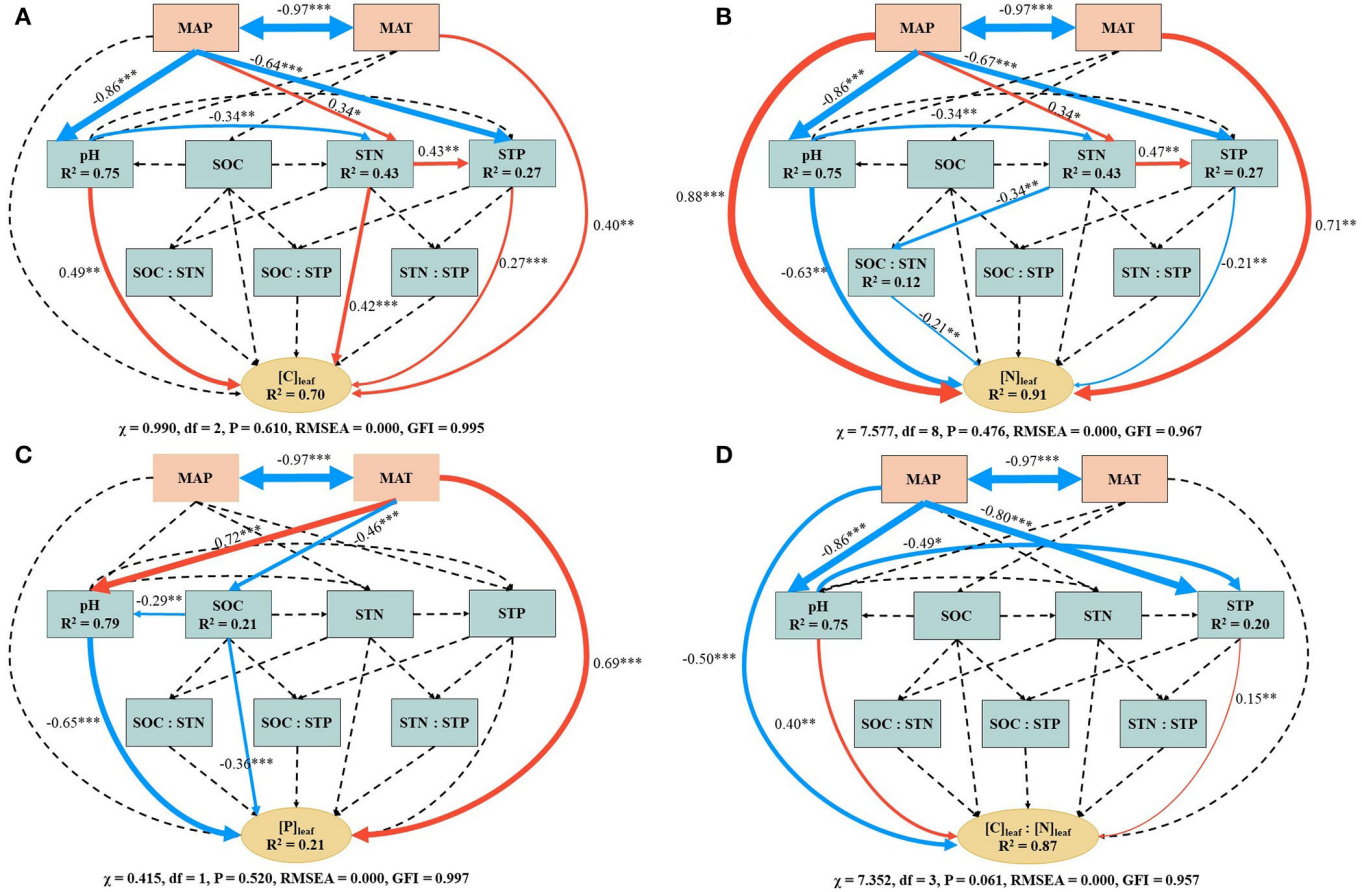
Structural equation modeling (SEM) examining the climatic and soil effects on leaf carbon concentration **(A)**, leaf nitrogen concentration **(B)**, leaf phosphorus concentration **(C)**, and the ratio of leaf carbon concentration and leaf nitrogen concentration **(D)**. [C]_leaf_, leaf carbon concentration; [N]_leaf_, leaf nitrogen concentration; [P]_leaf_, leaf phosphorus concentration; SOC, soil organic carbon; STN, soil total nitrogen; STP, soil total phosphorus; MAT, mean annual temperature; MAP, mean annual precipitation. Single- and double-headed arrows represent the hypothesized causal direction and covarying variables, respectively. Continuous and dashed arrows indicate the relationships with (*P < 0.05, **P < 0.01, ***P < 0.001) and without statistical significance (*P* > 0.05), respectively. Numbers next to continuous arrows are standardized path coefficients, which indicate the effect size of the relationship. Red and blue arrows indicate the positive and negative relationships, respectively, and arrow width is scaled proportionally to their effect size. *R*^2^ below variables indicates the proportion of variance explained.

The SEM showed that the climatic and soil factors accounted for about 70, 91, 21, and 87% of the total variation in [C]_leaf_, [N]_leaf_, [P]_leaf_, and [C]_leaf_/[N]_leaf_, respectively (Figure 3). Specifically, MAT had direct effects on [C]_leaf_, [N]_leaf_, and [P]_leaf_ and an indirect effect on [P]_leaf_ which may be from the positive influence on soil pH and negative influence on SOC (Figures 3A–C). MAP exerted direct effects on [N]_leaf_ and [C]_leaf_/[N]_leaf_ and indirect effects on [C]_leaf_, [N]_leaf_, and [C]_leaf_/[N]_leaf_ by influencing soil pH, STN, and STP (Figures 3A,B,D).

Soil pH and STP, which were both affected by MAP, exerted a direct positive effect on [C]_leaf_ and [C]_leaf_/[N]_leaf_ and a negative effect on [N]_leaf_ (Figures 3A,B,D). Soil pH also had a strong effect on [P]_leaf_ (Figure 3C). The STN had not only a direct effect on [C]_leaf_, but also an indirect effect as it positively influenced STP (Figure 3A). STN also indirectly affected [N]_leaf_ by negatively influencing SOC/STN and positively influencing STP (Figure 3B). The SOC had a direct negative effect on [P]_leaf_ and an indirect effect on [P]_leaf_ by negatively influencing soil pH (Figure 3C).

The SEM was not applicable for [C]_leaf_/[P]_leaf_ and [N]_leaf_/[P]_leaf_ because their models did not meet the requirement of P-values (*P* > 0.05). In this case, Pearson's correlation analysis was used to show their relationships with the environmental factors. Pearson's correlation analysis showed that the [C]_leaf_/[P]_leaf_ was only negatively correlated with MAT (*r* = −0.250, *P* < 0.05) and [N]_leaf_/[P]_leaf_ was positively correlated with MAP (*r* = 0.315, *P* < 0.05), but negatively correlated with MAT (*r* = −0.360, *P* < 0.01) and soil pH (*r* = −0.325, *P* < 0.01; Table 4).

**Table 4:** Pearson's correlations between environmental factors and leaf carbon-to-phosphorus ratio and the nitrogen-to-phosphorus ratio of *Potentilla anserina*.

	**MAT**	**MAP**	**Soil pH**	**SOC**	**STN**	**STP**	**SOC:STN**	**SOC:STP**	**STN:STP**
[C]_leaf_/[P]_leaf_	−0.250*	0.195	−0.197	0.118	0.067	−0.064	0.100	0.058	0.052
[N]_leaf_/[P]_leaf_	−0.360**	0.315*	−0.325**	0.176	0.154	−0.114	0.018	0.145	0.149

[C]_leaf_, leaf carbon concentration; [N]_leaf_, leaf nitrogen concentration; [P]_leaf_, leaf phosphorus concentration; SOC, soil organic carbon; STN, soil total nitrogen; STP, soil total phosphorus; MAT, mean annual temperature; MAP, mean annual precipitation. *P < 0.05, **P < 0.01.

## Discussion

### Effects of elevation on leaf ecological stoichiometry of *Potentilla anserina* and soil properties

The leaf stoichiometry of *P. anserina* changed due to heterogeneous habitat conditions with increased elevation. This agrees with the observations of van de Weg et al. (2009) and Hu et al. (2020) and supports our hypothesis that elevation would significantly influence leaf stoichiometry of *P. anserina*. However, Qin et al. (2022) found that elevation only significantly affected [C]_leaf_, [N]_leaf_, and [C]_leaf_/[N]_leaf_ of *Potentilla fruticosa*, while Su et al. (2022) reported that elevation was the key factor regulating the leaf stoichiometries of *Ligularia virgaurea* except [P]_leaf_ and [N]_leaf_/[P]_leaf_ in the Qilian Mountains. This suggests diverse adaptation strategies of different species to environmental changes.

The [C]_leaf_ of *P. anserina* showed a generally decreasing trend with an increase in elevation (Figure 2A), which is consistent with the study of Li et al. (2018) and Qin et al. (2022). This may be because photosynthesis was inhibited when the temperature dropped with an increase in elevation, which in turn weakened their carbon assimilation ability (Öquist, 1983). However, some studies (e.g., Zhao et al., 2018; Bo et al., 2020; Waigwa et al., 2020) reported that [C]_leaf_ tended to be greater at higher elevations to balance cell osmotic pressure and improve the frost resistance of plants (Zhao et al., 2018). These conflicting results suggest that the response of [C]_leaf_ to elevation still requires further studies based on species and at the regional level. The [N]_leaf_ is an important indicator of plant adaptation to environmental changes (Li et al., 2014). It showed a generally increasing trend with an increase in elevation (Figure 2B), and this agrees with the observations of Cao et al. (2020). This may be because *P. anserina* could increase [N]_leaf_ to offset the metabolic slowdown caused by a reduced enzymatic activity at low temperatures (McGroddy et al., 2004). The [P]_leaf_ of *P. anserina* showed a significantly decreasing trend with an increase in elevation (Figure 2C), and this is in line with the observations of Waigwa et al. (2020). In the study area, the variation of *P. anserina*'s [N]_leaf_ was consistent with the temperature–plant physiological hypothesis, while that of *P. anserina*'s [P]_leaf_ was in contrast to this hypothesis. This may be because of the decoupling of N and P in plants under global changes (Yuan and Chen, 2015). Therefore, it is necessary to conduct targeted research on the mechanisms for their changes with elevation separately. The [C]_leaf_/[N]_leaf_ and [C]_leaf_/[P]_leaf_ reflected the nutrient utilization efficiency, and a lower value indicated a higher plant growth rate and a lower N utilization efficiency (Elser et al., 2003; Weidner et al., 2015; Sun et al., 2017). In the Qilian Mountains, [C]_leaf_/[N]_leaf_ of *P. anserina* decreased with elevation, consistent with the study of Zhang Q. et al. (2019) and Zhang Y. et al. (2019), but contrary to the study of Hu et al. (2020), indicating that the growth rate of *P. anserina* increased but N use efficiency decreased with elevation (Guo et al., 2016; Zhang et al., 2020). This was consistent with the adaptive growth hypothesis that under N-limited environments, plants adopt a survival-first strategy and maintain higher C/N to increase N use efficiency and ensure survival, while under less N-limited environments, plants adopt a growth-first strategy and maintain lower C/N to keep higher growth rates (Zhang et al., 2020). In the study area, compared with lower elevations (i.e., 2,400–3,000 m), STN was relatively higher at higher elevations (i.e., 3,200–3,800 m) (Table 3), resulting in a less N-limited condition. Therefore, [C]_leaf_/[N]_leaf_ of *P. anserina* decreased at higher elevations to benefit growth priority. The [N]_leaf_/[P]_leaf_ of *P. anserina* increased with elevation, similar to the study of Cao et al. (2020) on *Oxytropis ochrocephala* and the study of Wang et al. (2019) on *Sabina przewalskii* in the Qilian Mountains, while Su et al. (2022) found no significant changes in *Ligularia virgaurea*'s [N]_leaf_/[P]_leaf_ across elevation in the Qilian Mountains. The increasing [N]_leaf_/[P]_leaf_ of *P. anserina* with elevation indicates that the growth of *P. anserina* in the study area may be more susceptible to P-limitation at higher elevations.

In the study area, soil properties differ significantly with elevation (Tables 1, 2). Similar results were reported by Cao et al. (2020) and Niu et al. (2021), who found contrasting soil nutrient contents with elevation in the Qilian Mountains. The differences in soil properties may be from the variations in solar radiation, temperature, and precipitation at different elevations (Qin et al., 2022). Zonal climatic conditions could change soil nutrient flux and soil nutrient allocation by affecting vegetation composition, litter quality, and the exchange of matter and energy between the soil and the environment (Jiang et al., 2019). Likewise, under the combined influence of various factors, soil pH and SOC also changed with elevation (Yu et al., 2019; Zhu et al., 2019).

### Mechanisms of elevation regulating leaf ecological stoichiometry of *Potentilla anserina*

As shown above, [C]_leaf_, [N]_leaf_, [P]_leaf_, and their ratios of *P. anserina* showed inconsistent responses to elevation (Table 3, Figure 2), similar to the study of Müller et al. (2017) and Tong et al. (2021), indicating that the mechanisms of elevation regulating leaf ecological stoichiometry of *P. anserina* were different. In order to figure out how elevation affects leaf ecological stoichiometry, the underlying mechanisms of the variation in each element under elevation need to be explored individually (Weiher and Keddy, 1999; Suding et al., 2008).

#### Mechanisms of elevation regulating [C]_leaf_ of *Potentilla anserina*

Temperature plays a key role in plant functions by influencing enzyme activity and membrane system fluidity as well as changing the absorption of nutrients and water and thus, in turn, affects the leaf ecological stoichiometry (Reich and Oleksyn, 2004; Hall et al., 2010; Liu et al., 2019). In the present study, MAT had a direct positive effect on [C]_leaf_ (Figure 3A), similar to the study of Fang et al. (2019). Previous researchers found that when MAT declined and photosynthesis weakened, plants increased leaf thickness and reduced leaf area in order to prevent freezing damage, finally leading to a decrease in [C]_leaf_ (Park and Day, 2007). With increasing elevation, the increased MAP can increase [C]_leaf_ by impacting soil pH, STN, and STP (Figure 3A).

The STN and STP exerted positive effects on [C]_leaf_ because high STN and STP were more conducive to the synthesis of enzymes that play key roles in carbon assimilation and accumulation (Zhang Q. et al., 2019). Soil pH exerted a positive influence on [C]_leaf_, consistent with the study of Liu M. et al. (2021). However, most previous studies showed that higher soil pH had a significant negative impact on [C]_leaf_ as it inhibits photosynthesis (e.g., He et al., 2016; Gong et al., 2018; Lin et al., 2022; Su et al., 2022). These results indicated that the impact of soil pH on [C]_leaf_ was significant but varied with species.

#### Mechanisms of elevation regulating [N]_leaf_ of *Potentilla anserina*

Elevation regulated [N]_leaf_ by changing MAT, MAP, soil pH, SOC/STN, STN, and STP (Figure 3B). Persson et al. (2012) found that lower MAT could reduce [N]_leaf_ by limiting the nutrient turnover rates. In the present study, MAT had a positive impact on [N]_leaf_ (Figure 3B), consistent with the study of Li et al. (2021). However, with the decrease in MAT, [N]_leaf_ showed a generally increasing trend (Table 3). This may be because the effect of MAT was weaker than the combined effects of MAP and soil physicochemical properties on [N]_leaf_. In general, MAP changed plant nutrient absorption and photosynthesis by affecting soil water content and soil nutrient availability (Han et al., 2011; Liu et al., 2019). In this study, MAP affected [N]_leaf_ either by directly changing its concentration, or by indirectly changing soil properties, further proved by the research of Reich (2005). MAP had a direct positive effect on [N]_leaf_, similar to the research of Sardans et al. (2005) and Chen Y. H. et al. (2013). This may be because, with the increase in MAP, the increase in soil water content could improve the plant photosynthetic rate and sucrose synthase and nitrate reductase activities, thus resulting in increased [N]_leaf_ (Patrick et al., 2009; Wang et al., 2011).

Decreased soil pH could promote the reabsorption of nitrogen to maintain plant growth, leading to increased [N]_leaf_ (Su et al., 2022). Haynes (1986) found that when SOC/STN was ≤ 25, soil nitrogen was mineralized as available nitrogen for plant growth, while when it was > 25, microorganisms mineralized soil nitrogen for maintaining their own growth. In the present study, the SOC/STN was all < 25, suggesting that more soil available nitrogen was taken up by plants and increased [N]_leaf_. However, this needs further research based on soil available nitrogen analysis. In addition, STP exerted a negative effect on [N]_leaf_. This might be because the decreased STP reduced the availability of P and relatively weakened the limiting effect of available N on plants, thereby enhancing the competition of plants for available N and promoting the uptake and utilization of N by an individual plant (Li et al., 2017). However, some studies found that decreased STP could reduce [N]_leaf_ (e.g., van Wijk et al., 2003; Mao et al., 2016). This was related to different responses of plant species to environmental changes due to their differentiation of niches based on functional attributes (Pontes et al., 2010; Adamidis et al., 2014; Li et al., 2017).

#### Mechanisms of elevation regulating [P]_leaf_ of *Potentilla anserina*

MAT had a positive effect on [P]_leaf_ (Figure 3C). This may be because the decrease in MAT restricted soil parent weathering, which led to a decrease in soil P absorption by plants (Tong et al., 2021). Besides, the decreased MAT can indirectly influence [P]_leaf_ through decreasing soil pH and increasing SOC with an increase in elevation. Soil pH had a direct negative impact on [P]_leaf_. This may be because the decrease in soil pH could release phosphate radicals and increase soil available P, thereby improving the uptake of P and [P]_leaf_ (Chen D. M. et al., 2013), while high soil pH may facilitate P adsorption to soil particles (Qiao et al., 2018).

The SOC had a negative effect on [P]_leaf_ (Figure 3C), contrary to what was observed by Liu et al. (2016) and Cao et al. (2020), suggesting that plants have their unique nutrient absorption mechanism in the processes of growth. The utilization of SOC by plants was extremely complex and may be controlled by a variety of soil physical and chemical properties (Pan et al., 2015; Rong et al., 2015).

#### Mechanisms of elevation regulating [C]_leaf_/[N]_leaf_/[P]_leaf_ of *Potentilla anserina*

According to the SEM, decreased [C]_leaf_/[N]_leaf_ with an increase in elevation resulted from the positive impacts of soil pH and STP and the negative impact of MAP (Figure 3D). This suggests that the increase in soil pH and STP is beneficial to promote the growth of *P. anserina* within a certain range, while the increase in precipitation may inhibit the growth of *P. anserina* in the study area. The [C]_leaf_/[P]_leaf_ at 3,800 m was significantly higher than that at 2,400, 3,200, and 3,500 m, similar to the study of Qin et al. (2022). However, there was no clear variation trend of [C]_leaf_/[P]_leaf_ with elevation (Figure 2E). This may be because the similar variations of [C]_leaf_ and [P]_leaf_ with elevation weakened the relationship between [C]_leaf_/[P]_leaf_ and elevation. MAT was negatively correlated with [N]_leaf_/[P]_leaf_ (Table 4), which might be attributed to the positive effect of MAT on [P]_leaf_ (Figure 3C), though MAT had a positive influence on [N]_leaf_. This indicated a stronger influence of MAT on [P]_leaf_ than on [N]_leaf_. The positive relationship between MAP and [N]_leaf_/[P]_leaf_ (Table 4) may be related to the positive effects of MAP on [N]_leaf_ (Figure 3B). However, the significantly increasing [N]_leaf_/[P]_leaf_ with elevation increase was mainly caused by significantly increasing [N]_leaf_ rather than by decreasing [P]_leaf_ in the study area.

### Other factors impacting leaf ecological stoichiometry of *Potentilla anserina*

According to the SEM results, the environmental factors in the present study can only account for part of the total variation of leaf ecological stoichiometry of *P. anserina* (Figure 3). This indicates the possibility of effects of other factors, such as inheritance and genetic mutations of species (Luo et al., 2006), vegetation community structure and composition (Wang et al., 2014; Zhu et al., 2020), light (Zhu et al., 2020), soil microbial activity (Li et al., 2014), and intra- and inter-species competitions (Xu et al., 2015; Qin et al., 2016). As *P. anserina* is a pioneer species of degraded grassland, grassland management also needs to be taken into consideration in future research to fully understand the leaf ecological stoichiometry changes of *P. anserina* and provide basic information for ecological restoration in this region.

### Nutrient limitation for *Potentilla anserina* growth across elevation

Due to the limited natural supply, N and P were the two most important elements to limit plant growth and functioning in a terrestrial ecosystem (Elser et al., 2000). Compared with the individual [N]_leaf_ and [P]_leaf_, [N]_leaf_/[P]_leaf_ was more reliable to identify plants' nutrient limitations (Li et al., 2018), although the thresholds were still controversial. In the present study area, the average [N]_leaf_/[P]_leaf_ (32.86) was significantly higher than that of global and Chinese vegetations (11.8 and 16.3, respectively) (Reich and Oleksyn, 2004; Han et al., 2005). This suggests that *P. anserina* was more P-limited than the averages of global and Chinese plants. According to the study of Güsewell (2004) on terrestrial plant communities, when [N]_leaf_/[P]_leaf_ < 10, plants growth was limited by N; 10 ≤ [N]_leaf_/[P]_leaf_ ≤ 20, plant growth was co-limited by N and P; and [N]_leaf_/[P]_leaf_ > 20, plant growth was limited by P. In the study area, the [N]_leaf_/[P]_leaf_ of *P. anserina* from 2,600 to 3,800 m was all > 20 and at 2,400 m was 10.05 (Table 3), suggesting that the growth of *P. anserina* was limited by P from 2,600 to 3,800 m and by both N and P at 2,400 m.

With increasing elevation, [N]_leaf_/[P]_leaf_ showed an increasing trend, indicating that the higher the elevation, the more severe the restriction of P element to the growth of *P. anserina*. This may be related to the decrease in MAT and soil pH and the increase in MAP (Tables 3, 4). Previous studies indicated that soil P deficiency was an important reason for P-limitation in plants in China (e.g., Han et al., 2005; Hu et al., 2017) and in the present study area (Cao et al., 2020; Qin et al., 2022). This also suggested that taking measures to increase soil available P and reduce P losses is of great importance for ecological restoration in the Qilian Mountains as well as in China.

## Conclusion

According to the results of field sampling and experimental analysis, [C]_leaf_, [N]_leaf_, [P]_leaf_, and the stoichiometric ratios of *P. anserina* fluctuated with elevation in the middle eastern part of the Qilian Mountains: from 2,400 to 3,800 m, [C]_leaf_, [P]_leaf_, and [C]_leaf_/[N]_leaf_ decreased, while [N]_leaf_ and [N]_leaf_/[P]_leaf_ increased. Elevation played a significant role in the leaf ecological stoichiometry of *P. anserina* through the effects of MAT, MAP, and soil pH. Based on [N]_leaf_/[P]_leaf_ inference, P element was the restrictive resource that affected *P. anserina* growth in the study area, and it was particularly deficient with the increase in elevation.

As *P. anserina* is a useful species for restoring degraded grasslands, it is of great importance to increase soil available P and reduce P losses for the growth of *P. anserina*, especially at higher elevations. Due to the relatively small scale of the present study, further studies in the Qilian Mountains will ensure wider applicability of the observations in other areas.

## Data availability statement

The original contributions presented in the study are included in the article/supplementary material, further inquiries can be directed to the corresponding author.

## Author contributions

XZ, QF, and JC were involved in the conception and design of the research. XZ and HS were involved in the acquisition of data and drafted the manuscript. XZ and YQ analyzed and interpreted the data. XZ and WL performed the statistical analysis. AB, JC, and MZ were involved in the revision of the manuscript for important intellectual content. All authors have read and approved the final manuscript.

## Funding

This work was supported by the National Natural Science Foundation of China (Grant No. 52179026), the Key R&D Program of Gansu Province, China (Grant No. 20YF8FA002), and the XPCC Science and Technique Foundation (Grant No. 2021AB021).

## Conflict of interest

The authors declare that the research was conducted in the absence of any commercial or financial relationships that could be construed as a potential conflict of interest.

## Publisher's note

All claims expressed in this article are solely those of the authors and do not necessarily represent those of their affiliated organizations, or those of the publisher, the editors and the reviewers. Any product that may be evaluated in this article, or claim that may be made by its manufacturer, is not guaranteed or endorsed by the publisher.
